# Exome sequencing confirms the clinical diagnosis of both joubert syndrome and klinefelter syndrome with keratoconus in a han Chinese family

**DOI:** 10.3389/fgene.2024.1417584

**Published:** 2024-07-15

**Authors:** Xinhe Fang, Meijiao Ma, Weining Rong, Yuan-Yuan Lian, Xueli Wu, Yongying Gao, Hui-Ping Li, Xunlun Sheng

**Affiliations:** ^1^ Ningxia Eye Hospital, People’s Hospital of Ningxia Hui Autonomous Region, Third Clinical Medical College of Ningxia Medical University, Yinchuan, China; ^2^ Gansu Aier Ophthalmology and Optometry Hospital, Lanzhou, China

**Keywords:** keratoconus, joubert syndrome, klinefelter syndrome, *CPLANE1* gene, variation

## Abstract

**Introduction:**

Joubert syndrome a rare genetic disorder, is characterized by abnormalities in the development of the central nervous system with “molar signs” on magnetic resonance imaging of the brain and accompanied by cerebellar vermis hypoplasia, ataxia, hypotonia, and developmental delay. Keratoconus (KC) is a kind of genetically predisposed eye disease that causes blindness characterized by a dilated thinning of the central or paracentral cornea conically projected forward, highly irregular astigmatism, and severe visual impairment. Klinefelter syndrome is caused by an extra X chromosome in the cells of male patients, and the main phenotype is tall stature and dysplasia with secondary sex characteristics. This study was intended to identify the genetic etiology and determine the clinical diagnosis of one Han Chinese family with specific clinical manifestations of keratoconus and multiorgan involvement.

**Methods:**

A comprehensive ocular and related general examination was performed on one patient and his asymptomatic parents and brother. Pathogenic genes were tested by exome sequencing. CNV-seq was used to verify the copy number variation, and peripheral blood was cultured for karyotype analysis. The pathogenicity of the identified variant was determined subject to ACMG guidelines. The Gene Expression Omnibus (GEO) dataset of keratoconus-related genes in the NCBI database was obtained to analyze the differentially expressed genes in corneal tissues of the keratoconus group and the normal control group, and analysis of protein-protein interaction networks (PPI) was performed.

**Results:**

Proband, a 25-year-old male, had sudden loss of vision in the left eye for 1 week. Best corrected visual acuity (BCVA): 0.5 (−1.00DS/-5.00DC*29°) in the right eye, counting fingers/40 cm in the left eye. Slit-lamp microscopy of the right eye showed mild anterior protrusion of the cornea and thinning of the cone-topped cornea. The left eye showed marked thinning of the central region of the cornea, rounded edema in the form of a cone-like bulge, epithelial bullae, edema and turbidity of the stroma, and bulging of the Descemet’s membrane. Cranial magnetic resonance imaging (MRI) revealed changes in the midbrain and cerebellum, with a “molar sign” and a “bat-winged” ventriculus quartus cerebri. General check-up: 168 cm in height, decreased muscle tone in all four limbs, knee jerk elicited, negative Babinski sign, abdominal reflexes elicited, finger-to-nose test positive, intentional tremor evident in both hands, positive Romberg’s sign, instability of gait, level I intellectual disability, poor adaptive behavior, communication disorders, teeth all dentures, a peculiar face with blepharophimosis, wide inner canthus distance, mild ptosis, severe positive epicanthus, high palatal arches, exotropia, hypotrichosis of beard and face, inconspicuous prominentia laryngea, and short upper and lower limbs. Exome sequencing detected compound heterozygous frameshift variants M1:c.9279dup:*p*.His3094Thrfs*18 and M2:c.6515_6522del:*p*.Lys2172Thrfs*37 in the patient’s *CPLANE1* gene and the presence of duplication-type CNV on the X chromosome. Sanger sequencing showed that the mother and father carried the M1 and M2 variants, respectively, and the younger brother carried the M2 variant, which was a novel variant. CNV-seq analysis showed the presence of a duplication-type CNV Xp22.33-Xq28 (2757837-156030895) of approximately 155 Mb on the X chromosome of the proband, which was a *de novo* variant and carried by neither of the parents. The two heterozygous frameshift variants and duplication-type CNV were pathogenic according to the ACMG guidelines. Differential expression analysis of keratoconus-related genes showed that *CPLANE1* was upregulated in the corneal tissues of keratoconus patients compared with normal controls, and such a difference was statistically significant (*p* = 0.000515, <0.05). PPI analysis showed that the CPLANE1-NPHP3 complex protein acted as a bridge between cilia and extracellular matrix tissue. According to the genetic test results and clinical phenotype analysis, the family was finally diagnosed with Joubert syndrome combined with Keratoconus and Klinefelter syndrome.

**Discussion:**

In this study, we report a proband in a Han Chinese family with both Joubert syndrome and X-linked Klinefelter syndrome as well as keratoconus, and the phenotype spectrum of *CPLANE1*-Joubert syndrome may be expanded accordingly. Meanwhile, the significance of exome sequencing was emphasized in aiding the clinical diagnosis of complex cases, which is difficult to make.

## 1 Introduction

Joubert syndrome (JS) is a rare genetic disorder with an incidence of 1:80,000-1:100,000 (Brancati et al., 2010). Joubert syndrome has a variety of clinical manifestations, mainly characterized by abnormalities in the development of the central nervous system, including hypotonia, abnormal respiratory rhythm, oculomotor disorders, ataxia, and developmental delay (Valente et al., 2013), and may also be combined with other manifestations. Maria et al. (Maria et al., 1997) found that there was a common abnormality in the cranial MRI of the JS patients, i.e., patients with cerebellar vermis hypoplasia showed the phenomenon of “molar tooth sign” at the junction between midbrain and hindbrain in the MRI. Subsequently, the “molar tooth sign” shown in the MRI was taken as a characteristic diagnostic marker for Joubert syndrome. The clinical heterogeneity of JS is closely related to its significant genetic heterogeneity. To date, at least 35 genes have been identified to be associated with JS (Vilboux et al., 2017), most of which are inherited in an autosomal recessive mode including *TMEM67, CPLANE1* (NM_023073)*, CC2D2A*, etc. Only one rare gene, *OFD1*, shows X-linked recessive inheritance (Parisi, 2019). The *CPLANE1* gene, also known as *C5ORF42*, is located on chromosome 5p13.2 and is a cilium gene that encodes the ciliogenesis and planar polarity effector complex subunit 1 protein. Abnormalities in the *CPLANE1* gene can result in autosomal recessive Joubert syndrome type 17 (OMIM#614615) or oral-facial-digital syndrome type 6 (OMIM#277170) (Poretti et al., 2017). *CPLANE1*-JS patients had mostly a purely neurologic phenotype, with a few presenting with polydactyly. None of them showed renal, hepatic, or retinal disease, and intellectual disability was less severe than in patients with other types of JS (Enokizono et al., 2017). Moreover, neuroimaging changes were less severe than in other types of JS, which made it easy to result in underdiagnosis.

Keratoconus (KC) is one of the most important genetically predisposed eye diseases that severely jeopardizes the visual function of adolescents and causes blindness characterized by a dilated thinning of the central or paracentral cornea conically projected forward, highly irregular astigmatism, and severe visual impairment. The pathogenesis of keratoconus remains unclear yet. With the development of genetics and genetic testing techniques, more and more studies have confirmed the role of genetic factors in the pathogenesis of KC (Liyan et al., 2020). At present, it is believed that the main inheritance modes of keratoconus are autosomal recessive and dominant inheritance as well as mitochondrial inheritance. Genetic studies of KC-associated genes using mutational analysis of pathogenic genes showed that the occurrence of KC has significant genetic heterogeneity, and the defects of numerous genes in the nuclear genome can lead to the occurrence of KC. Currently, 25 KC-related pathogenic genes have been identified (Hashemi et al., 2020). A study in 2022 adopted target sequence capture combined with high-throughput sequencing technology to detect 6 *de novo* variants in such genes as *HMX1*, *SLC4A11, TGFBI, PIKFYVE,* and *ZEB1* in 5 KC families, confirming that the pathogenic variants in these 5 genes are associated with the occurrence of KC (Cheng et al., 2022). However, the genes known to be associated with the pathogenesis of KC can only explain part of the pathogenesis. The pathogenesis of many KC patients cannot be explained by known genes, indicating that there are still a large number of unknown genes to be discovered.

With the deepening of genetic research in recent years, scholars have studied the common target tissue, ciliary dysfunction, caused by different gene mutations encoding cilia-centrosome complex-related proteins, as a class of diseases and put forward the concept of “ciliopathy.” Cilia are hair-like projections found on almost all cells in the human body and play an important role in the formation and maintenance of the body’s tissues and organs. Abnormalities in ciliary structure and function affect almost every system of the body, such as the brain, eyes, liver, kidneys, bones, and reproductive system. Ciliary dysfunction associated with visual development can be categorized into simple (non-syndromic) ciliopathies and syndromic ciliopathies that combine with other systemic diseases. In studies of retinal ciliopathy, it has been found that Bardet-Biedl syndrome (BBS) (Weihbrecht et al., 2017) and Leber congenital amaurosis (LCA) (Skorczyk-Werner et al., 2020) are often accompanied by KC.

Klinefelter syndrome is one of the most common chromosomal disorders, with an incidence of 0.1%–0.2% in males, accounting for 3%–4% of infertile males, and 10%–12% in azoospermia patients (Flannigan et al., 2018). In 1942, Klinefelter et al. (Klinefelter, 1986) described a male hypogonadism syndrome characterized by increased secretion of follicle-stimulating hormone (FSH) and first introduced the concept of Klinefelter syndrome. The karyotypes of Klinefelter syndrome were later found to include 47, XXY, 48, XXXY, 48, XXYY, 49, XXXXY, etc., among which the karyotypes 47, XXY accounted for about 88% (Linden et al., 1995). Klinefelter syndrome progressively worsens behavioral and cognitive deficits as the number of X chromosomes increases. The phenotype of some patients is devoid of any clinically significant symptoms other than small testicular size, and only about 10% of patients with Klinefelter syndrome can be diagnosed before puberty (Groth et al., 2013).

This paper reports a patient from a non-consanguineous married healthy Chinese family. The patient was presented to the ophthalmology department with binocular vision loss and was diagnosed with keratoconus, and was also found to be associated with Joubert syndrome (JS) and Klinefelter syndrome-related clinical phenotypes. The exome sequencing was performed to detect genetic variants, identify pathogenic genes, and explore genotypic and clinical phenotypic features of Joubert syndrome-associated ocular disorders to provide a reliable molecular diagnosis of KC-JS and to help clinicians improve their understanding of the disease.

## 2 Objects and methods

### 2.1 Objects

A proband with keratoconus and a complex clinical phenotype of multi-organ involvement from a healthy family was collected from Gansu Aier Ophthalmology and Optometry Hospital. Detailed inquiries were made about growth and development, family history, marriage and parenthood, and the history of systemic diseases, and a family tree was drawn. Patient and family members underwent the examination, including uncorrected visual acuity (UCVA), Best corrected visual acuity (BCVA), slit lamp microscope with front mirror, color fundus photography (CFP, TRC-NW300, TOPCON, Japan), panoramic Ophthalmoscope-Daytona (P200T, United Kingdom), optical coherence tomography (OCT, HD-OCT4000, Carl Zeiss Meditec, United States), anterior segment analysis system (Pentacam 70,700, Germany), and corneal biomechanics analyzer (Corvis ST 72100, Germany). The general examination comprised abdominal color ultrasound, cranial MRI, limb length measurements, and intelligence tests. The full length of the upper limb is the distance from the acromion to the styloid process of the radius. The total length of the lower limb is the distance from the anterosuperior iliac spine to the medial malleolus of the tibia.

In this study, the diagnostic criteria and staging criteria for keratoconus as specified in the Chinese Expert Consensus on Diagnosis and Treatment of Keratoconus (2019) were adopted. The diagnostic criteria for Joubert syndrome include: 1) “molar sign” caused by cerebellar vermis hypoplasia on imaging; 2) decreased muscle tone; 3) developmental delay or intellectual disability; 4) abnormal breathing and/or abnormal eye movements (unnecessary but suggestive). Klinefelter syndrome is caused by an extra X chromosome in male patients, so karyotyping is the standard diagnosistic method for Klinefelter syndrome.

The study project was approved by the Ethics Committee of the Gansu Aier Ophthalmology and Optometry Hospital [Approval No.: GSAIER2023IRB03] and was in strict compliance with the Declaration of Helsinki. For one subject who was not competent to give informed consent, his or her guardians were informed and signed written informed consent.

### 2.2 Methods

#### 2.2.1 Genomic DNA extraction

6 mL of peripheral venous blood was collected from the patient and family members and anticoagulated with EDTA. The whole genomic DNA was extracted, and the genomic library was constructed using the Qiamp Blood Mini Kit DNA extraction kit from QIAGEN, Germany, in accordance with the operating procedures (DNA mass concentration ≥50 ng/μL, total DNA ≥6 μg).

#### 2.2.2 Exome sequencing

Whole-genome exome capture was performed using the SureSelect Exon Capture Kit from Agilent: Target gene exons and adjacent splice regions (about 20 bp), as well as the full length of the mitochondrial genome, were captured by probe hybridization and enriched. The enriched genes were subjected to quality control, and high-throughput sequencing was performed using the Illumina NextSeq 500 platform of Illumina (United States), with a sequencing depth of 100×. Illumina Basecalling Software 1.7 was used to compare the raw sequencing reads with the Human Genome DNA Reference Sequence (NCBI Build 37.1) provided by the National Center for Biotechnology Information (NCBI). GATK and HaplotypeCaller were used to analyze the information related to single nucleotide variants (SNV) and insertion and deletion variants (InDel), respectively, to obtain all the variant sites. High-frequency variants with minimum allele frequency (MAF) values > 1% and variants with no effect on protein function and structure were screened. The candidate pathogenic gene variants were obtained and stepwise screened. Co-segregation analysis was performed among members of the patient’s family using Sanger sequencing.

#### 2.2.3 Copy number variation sequencing (CNV-seq)

Twist Library Preparation EF Kit 2.0 (104207) was used for library construction. The library was sequenced by DNBSEQ-T7 platform. The sequencing data were compared with the human genome reference sequence hg38 by BWA. CNVkit was applied to detect copy number variations (CNVs) (Talevich et al., 2016). Candidate CNVs were filtered with the Database of Genome Variants (DGV) (http://dgv.tcag.ca/dgv/app/home) and NCBI (https://www.ncbi.nlm.nih.gov/). Decipher (https://decipher.sanger.ac.uk/). ClinVar (https://www.clinicalgenome.org/), ClinGen (https://www.clinicalgenome.org/), and OMIM (https://www.omim.org/) were included to annotate the genes or genomic regions in the candidate pathogenic CNVs.

#### 2.2.4 Karyotype analysis

High-resolution G-banding karyotype analyses were performed using trypsin digestion method and Giemsa staining method. A total of 10 metaphase cells were analyzed. Karyotype summaries were made according to the International System for Human Cytogenetic Nomenclature.

#### 2.2.5 Analysis of variation in pathogenicity

The pathogenicity of novel variants and CNV was assessed in pathogenicity according to Standards and Guidelines for Interpretation of Sequence Variants issued by the American College of Medical Genetics and Genomics (ACMG) in 2019 (Riggs et al., 2020). MAF <0.005 was used as the criteria to exclude benign variants by reference to the databases for East Asian populations with allele frequencies available with the 1000 Genomes Project (1000G, http://browser.1000genomes.org) and ExAC Browser (http://exac.broadinstitute.org/).

#### 2.2.6 Genetic expression and functional analysis

The Gene Expression Omnibus (GEO) dataset relating to keratoconus (GSE77938) (Kabza et al., 2017) was obtained from the NCBI database, including 25 keratoconus and 25 non-keratoconus from RNA sequencing of human corneas. Analyzing with GEO2R, a built-in online analysis tool of the GEO database (https://www.ncbi.nlm.nih.gov/geo/info/geo2r.html), was used to obtain the keratoconus-related differentially expressed genes (DEGs), and the result was plotted on the volcano plot. Genes with both high fold change and statistical significance are typically represented as points located towards the upper ends (upregulated genes) or lower ends (downregulated genes) of the plot, resembling the peaks of a volcano. The STRING database (https://string-db.org) was used to detect gene interactions, which integrates known and predicted protein-protein interaction networks (PPIs), and protein interactions with a confidence coefficient greater than 0.4 were selected for further analysis. Proteins were clustered by K-mean clustering in String. ClusPro Server (https://cluspro.org) was used for protein-protein docking. The SWISS-MODEL template library (SMTL version 2024-06-05, PDB release 2024-05-31) was searched for evolutionary-related structures matching the target sequence in NPHP3 and CPLANE1. The NPHP3 template is Q7TNH6.1. A, the AlphaFold DB model is NPHP3_MOUSE, the seq identity is 88.97%, the coverage is 100%, and the and the GMQE is 0.72. CPLANE1 template is D3ZK35.1. A, AlphaFold DB model is D3ZK35_RAT, seq identity is 71.49%, coverage is 80%, and GMQE is 0.43. Uploaded the PDB file of the protein structure to ClusPro Server to get the docking model. The 3D structure of protein-protein docking was visualized using PyMOL.

#### 2.2.7 Literature review

A search was conducted on PubMed using“*CPLANE1*,” “Joubert syndrome,” “Mutation,” “exon” as the terms to search the relevant literature for analyzing the relationship between *CPLANE1* genotypes and clinical phenotypes as shown in Table 2.

## 3 Results

### 3.1 Physical data

Proband, a 25-year-old male, presented to the hospital with a sudden loss of vision in the left eye without any apparent cause, accompanied by a foreign body sensation, eye irritation, photophobia, and tearing. His parents were not consanguineous and denied any family history of disease. Inquiry about birth and medical history: The patient was born at term, weighing 3.0 kg at birth, with no abnormal appearance and no pathologic jaundice at birth. The prenatal examination was normal, and the fetus had no signs of intrauterine distress. He was able to sit unaided for over 3 years, walk unaided at the age of 4, and speak at the age of 3, but his expression was slurred. The patient had no convulsions.

### 3.2 Ophthalmic examinations

**FIGURE 1:**
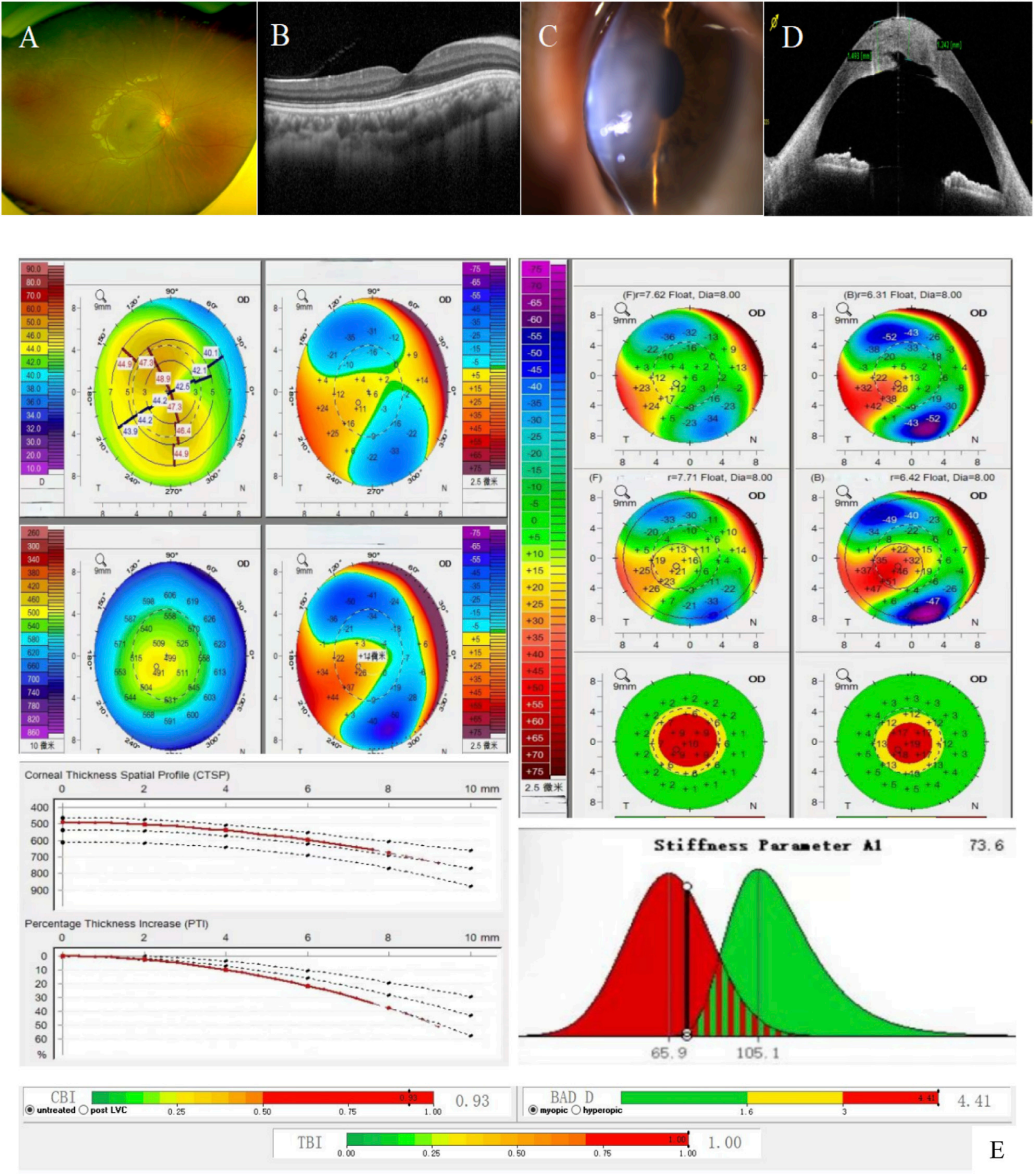
Patient’s ophthalmologic examination, corneal topography map, and corneal biomechanics examination: **(A)** and **(B)**. Fundus photography and macular OCT of the right eye showed no obvious abnormalities; **(C)**. Anterior segment image of the left eye: obvious thinning of the central area of the cornea, rounded edema in the form of a cone-like bulge, epithelial bullae, edema and opacity of the stromal layer, descemetocele; **(D)**. OCT of the anterior segment of the left eye: corneal epithelium intact with a subepithelial effusion; edematous corneal stroma with an increase in thickness; and broken and discontinuous Descemet’s membrane; **(E)**. Corneal topography map and corneal biomechanics of the right eye: Df, Db, Dp, D, BAD-D are pathologic values (red); Da was suspicious value (yellow); corneal thickness was thinner (491 μm in the right eye); K1 was 43.4 D and K2 was 48.0 D (a difference of 4.6 **(D)**; SP-A1 was <105.2 (73.6 in the right eye): the smaller the value, the greater the risk of keratectasia.

Proband suffers from concomitant exotropia. BCVA: 0.5 (−1.00DS/-5.00*29°) in the right eye, counting fingers/40 cm in the left eye. Intraocular pressure in the right eye: 14.9 mmhg, undetectable in the left eye. Slit-lamp microscopy of the right eye showed mild anterior protrusion of the cornea, thinning of the cone-topped cornea, and no obvious abnormality in the remaining anterior segment of the eye, the lens, and the fundus (Figures 1A,B); Slit-lamp microscopy of the left eye showed congestion of the bulbar conjunctiva++, obvious thinning of the central area of the cornea, rounded edema in the form of a cone-like bulge, epithelial bullae, edema and opacity of the stromal layer, descemetocele, and the inability to see into the lens and the fundus (Figures 1C,D). Pentacam 3D anterior segment analysis and Corvis ST on the right eye (Figure 1E) suggested that the flat keratometry K1 at 3 m from the center of the cornea was 43.4 D, the steep keratometry K2 was 48.0 D, the max keratometry (Kmax) of the anterior surface was 49.0 D, the thinnest point thickness (TP) was 491 μm, and the deviation of normality of the front elevation (Df) was 4.77 SD (standard deviation), the deviation of normality of the back elevation (Db) was 3.32 SD, the deviation of normality of pachymetric progression (Dp) was 3.13 SD, the deviation ofnormality ofcorneal thinnest point (Dt) was 1.49 SD, the deviation of normality of relational thickness (Da) was 1.74 SD, and the Belin/Ambrósio enhanced ectasia display final D value (BAD-D) was 4.41, which reflects the overall risk profile of keratoconus (<1.6SD for normal values of Df, Db, Dp, Dt, Da, 1.6–2.6 SD for suspicious values (yellow), and >2.6 SD for pathologic values (red)). Several testing and monitoring indicators of corneal biomechanics analyzed by Belin were pathological values: Corvis biomechanical index CBI was 0.93 [0.25–0.5 for suspicious values (yellow) and >0.5 for pathological values (red)]; Tomographic and biomechanical index TBI was 1.00 (0.25—0.75 for suspicious values (yellow), and >0.75 for pathologic values (red)); and SP-A1 was 73.6 (<105.2, the smaller the value, the greater the risk of corneal dilatation). The left eye was not measured for topography-related values due to corneal edema. The right eye was diagnosed clinically as keratoconus (completion period, grade 1) and the left eye as acute keratoconus.

Pentacam corneal topography map, Belin analysis, and corneal biomechanical analysis were performed on the patient’s parents and brother, respectively (see Table 1). No significant abnormality was found in the father (I-1) (Supplementary Figure S1). The mother (I-2) had a pathologic value of 4.80 SD for the deviation of normality of the front elevation (Df), a suspicious value of 2.05 SD for the deviation of normality of pachymetric progression (Dp), an SP-A1 of 65.6, a BAD-D value of 2.69, and a suspicious value of 1.74 SD (right) and 1.94 SD (left) for the deviation of normality of the back elevation (Db) in the left corner, which showed a microcorneal morphology. The deviation of normality of pachymetric progression (Dp) in the younger brother (II-2) was suspicious value of 1.84 SD (right) and 1.99 SD (left).

**TABLE 1:** Keratectasia and corneal biomechanical indices in proband and family members.

Patient		Ⅱ-1	Ⅱ-2	Ⅰ-1	Ⅰ-2
Sex		M	M	M	F
Age		25	9	52	49
BCVA	OD	0.5 (−1.00DS/-5.00DC*29°)	1.0 (−0.50DS/-0.25DC*118°)	1.0 (-0.50DS/-0.50DC*77°)	1.0 (-0.75DS/-2.25DC*95°)
	OS	counting fingers/40 cm	1.0 (−0.50DS)	1.0 (−0.50DC*99°)	1.0 (−2.25DC*99)
K1(D)	OD	43.4	43.0	45.9	42.8
	OS	NA	43.0	45.9	42.8
K2(D)	OD	48.0	43.7	46.4	44.2
	OS	NA	43.5	46.5	44.2
K_max_(D)	OD	49.0	44.3	46.8	44.5
	OS	NA	44.3	47.1	44.7
TP (μm)	OD	491	538	512	530
	OS	NA	522	507	522
Df (SD)	OD	4.77	1.35	1.36	1.00
	OS	NA	1.43	1.34	4.80
Db(SD)	OD	3.32	0.28	0.72	1.74
	OS	NA	0.77	1.38	1.94
Dp (SD)	OD	3.13	1.84	0.51	1.24
	OS	NA	1.99	1.46	2.05
Dt (SD)	OD	1.49	−0.01	0.78	0.24
	OS	NA	0.46	0.94	0.47
Da (SD)	OD	1.74	0.91	0.40	1.12
	OS	NA	1.05	1.25	1.17
CBI	OD	0.93	0.06	0.13	0.04
	OS	NA	0.00	0.00	0.01
TBI	OD	1.00	0.57	0.20	0.34
	OS	NA	0.47	0.32	1.00
SP-A1	OD	73.6	92.8	89.9	93.2
	OS	NA	96.2	93.2	65.6
BAD-D	OD	4.41	1.56	1.54	1.93
	OS	NA	1.73	2.30	2.69

Red highlights are pathologic values, and yellow highlights are suspicious values.

The patient was admitted to the hospital and underwent thermokeratopkty in the left eye and corneal collagen cross-linking in the right eye, respectively. BCVA was reviewed 40 days after surgery: 0.4 (−0.75DS/-6.50DC*30°) for right eye, counting fingers/10 cm for left eye; intraocular pressure: 16.7 mmhg in the right eye, 18 mmhg in the left eye; transparent cornea was seen in the right eye under the slit-lamp microscope with a smooth epithelium and KP(−); Slit-lamp microscopy of the left eye showed mild corneal edema, conical anterior protrusion of the central cornea, obvious edema of the corneal stroma, descemetocele, Fleischer’s ring (+), Vogt’s (+), Munson’s sign (−), and KP (−). Penetrating keratoplasty was performed in the left eye 1 year later, and the BCVA of the left eye was reexamined 2 weeks after surgery: 0.5 (+0.25/+2.50*10°), IOP of the left eye: 18.5 mmhg, and the slit-lamp microscopy showed translucent corneal graft, graft planting bed in good alignment, sutures in place, and no anterior protrusion of the cornea.

### 3.3 General check-up

**FIGURE 2:**
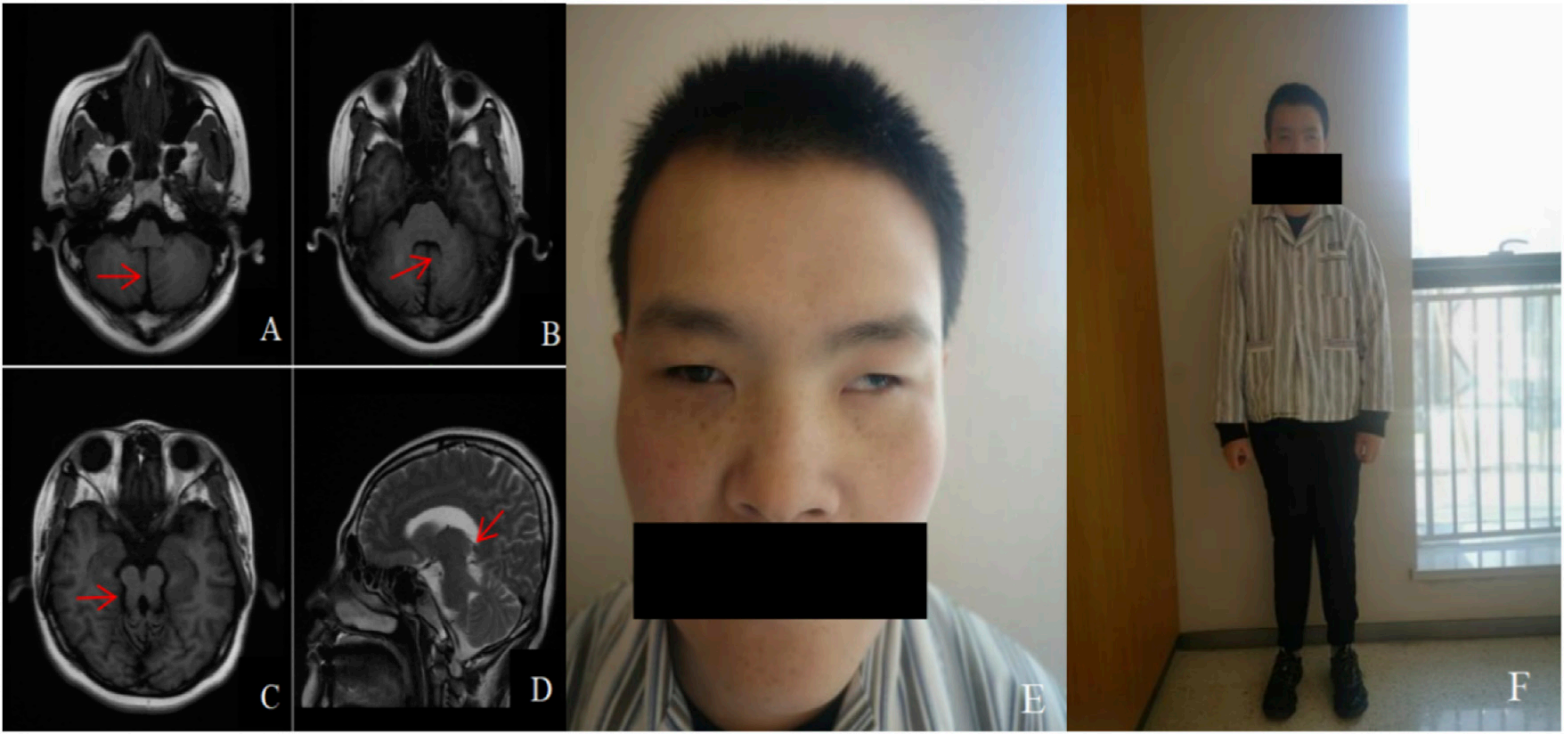
Cranial MRI and facial and systemic manifestations of patient: **(A)**. Axial view T1WI shows the “midline cleft” sign (red arrow); **(B)**. Axial view T1WI shows the “batwing” fourth ventricle (red arrow); **(C)**. Axial view T1WI shows the “molar sign” (red arrow); **(D)**. Sagittal view T2WI shows the thickened and elongated pedunculus cerebellaris superior, which runs in an anterior-posterior direction (red arrow); **(E)**, **(F)**. Special face, featuring blepharophimosis, wide inner canthal distance, mild ptosis, severe positive epicanthus, high palatal arches, exotropia, hypotrichosis of the beard and face, and inconspicuous prominentia laryngea; Fair skin with no fine hair all over, shorter upper and lower limbs, and instability of gait.

The patient was 168 cm in height and 65 kg in weight (the standard weight is 59 kg for a normal 25-year-old adult male with a height of 168 cm). He had a peculiar face with blepharophimosis, a wide inner canthal distance, mild ptosis, severe positive epicanthus, high palatal arches, exotropia, hypotrichosis of the beard and face, and inconspicuous prominentia laryngea (Figure 2E). His upper limb was 70 cm and his lower limb was 89 cm [in Nanyang Han adult males, the upper and lower limb length indices are 74.16 cm and 92.13 cm, respectively (Ying et al., 2013)]. All the teeth were dentures. Reduced muscle tone in all limbs, knee jerk elicited, negative Babinski sign, abdominal reflexes elicited, finger-to-nose test positive, intentional tremor evident in both hands, positive Romberg’s sign, instability of gait (Figure 2F). No hearing abnormalities, steady breathing, no polydactyly, no single transverse palmar crease, and fair skin with no fine hair all over. According to the World Health Organization (WHO) and American Association for Mental Retardation (AAMD) classification standards for intellectual disability, the grades were divided according to intelligence quotient (IQ) and social adaptive behavior. The patient’s IQ < 20, poor adaptive behavior, and communication disorders belong to level I intellectual disability. Cranial magnetic resonance imaging (MRI) showed changes in the midbrain and cerebellum, with a “molar sign” and a “bat-wing” fourth ventricle (Figures 2A–D). Abdominal ultrasonography, blood and urine routine analysis, and liver and kidney function tests showed no abnormalities.

### 3.4 Genetic testing and pathogenicity analysis

Exome sequencing revealed the proband carried novel compound heterozygous frameshift variants M1:c.9279dup (*p*.His3094Thrfs*18) and M2:c.6515_6522del (*p*.Lys2172Thrfs*37) in the *CPLANE1* gene. M1 was a base duplication at site 9,279 of cDNA, resulting in a change of codon 3,094 from histidine encoded to threonine, followed by a frameshift to advance the termination codon, which likely leads to nonsense-mediated mRNA degradation; M2 was a deletion of bases 6,515 to 6,522 of cDNA, resulting in a change of codon 2,172 from lysine encoded to threonine, followed by a frameshift to advance the termination codon, which likely leads to nonsense-mediated mRNA degradation. Both M1 and M2 variants in the *CPLANE1* gene were frameshift variations, which may trigger nonsense-mediated decay (NMD) of an altered transcript and lead to a complete absence of the gene product (PVS1_Very Strong).

Both M1 and M2 variants were rare variants that have not been previously reported in the related literature and were also not detected in the gnomAD genomes database or East Asian Population Database (ExAC-EAS) (PM2_Moderate). Further verification in other family members (I:1, I:2, and II:2) by Sanger sequencing showed that the normal mother and father carried the M1 and M2 variants, respectively, and the normal younger brother carried M2, suggesting that the genotype and the clinical phenotype were co-separated (PP1). Both M1 and M2 variants were pathogenic according to ACMG guidelines. Taken together, these novel compound heterozygous frameshift variants in *CPLANE1* are more likely to cause KC-Joubert syndrome by affecting the function of the *CPLANE1* protein.

**FIGURE 3:**
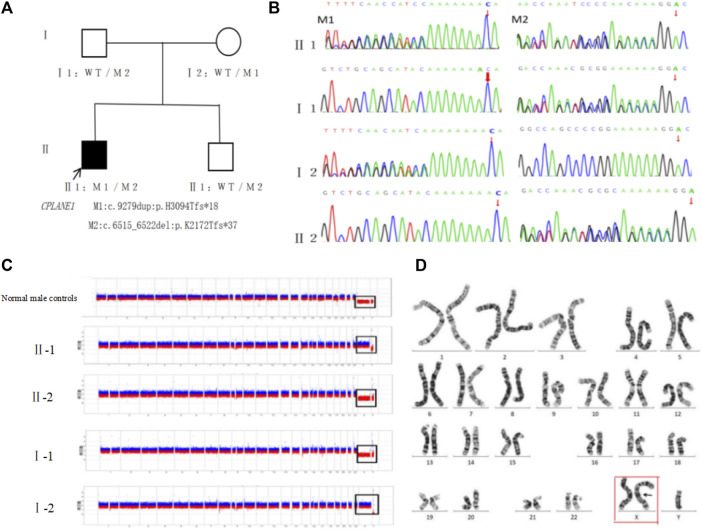
Genetic testing and pathogenicity analysis of the proband: **(A)**. Family tree: *CPLANE1* variant loci M1:c.9279dup:*p*.His3094Thrfs*18 and M2:c.6515_6522del:*p*.Lys2172Thrfs*37 were co-segregated in the family between the patient and the normal phenotypic members. **(B)**. Sanger sequencing diagram: the father of the proband carried M2, the mother carried M1, the younger brother carried M2 but failed to carry M1, and such two variants were compound heterozygous variants. **(C)**. Genetic testing of CNV: the proband (II-1) had duplication-type CNV Xp22.33-Xq28 (2757837-156030895) of ∼155 Mb on chromosome X, including 240 known OMIM pathogenic genes, suggesting that there is an extra copy of the entire X chromosome; the parents (Ⅰ-1, Ⅰ-2) and the younger brother (Ⅱ-2) did not show any obvious pathogenic CNV. **(D)**. Peripheral blood karyotyping confirmed the karyotype to be 47, XXY (red box).

At the same time, exome sequencing revealed that the proband also had a duplication-type CNV on the X chromosome. The CNV-seq test confirmed that the proband had about 155 Mb of duplication-type CNV Xp22.33-Xq28 (2757837-156030895) on the X chromosome, including 240 known OMIM pathogenic genes such as *BCOR, HCCS, GATA1*, and so on, suggesting that there is an extra copy of the entire X chromosome. Such a variant was a novel one that neither the parents nor the younger brother carried (Figure 3C). The variant was determined to be pathogenic according to the ACMG guidelines. Chromosome karyotype analysis was performed on peripheral blood after culture (400 bands, G banding), and it was confirmed that the karyotype was 47, XXY, which was the karyotype of Klinefelter’s syndrome (Figure 3D).

### 3.5 Analysis of differentially expressed genes and protein-protein interactions of the *CPLANE1* gene

**FIGURE 4:**
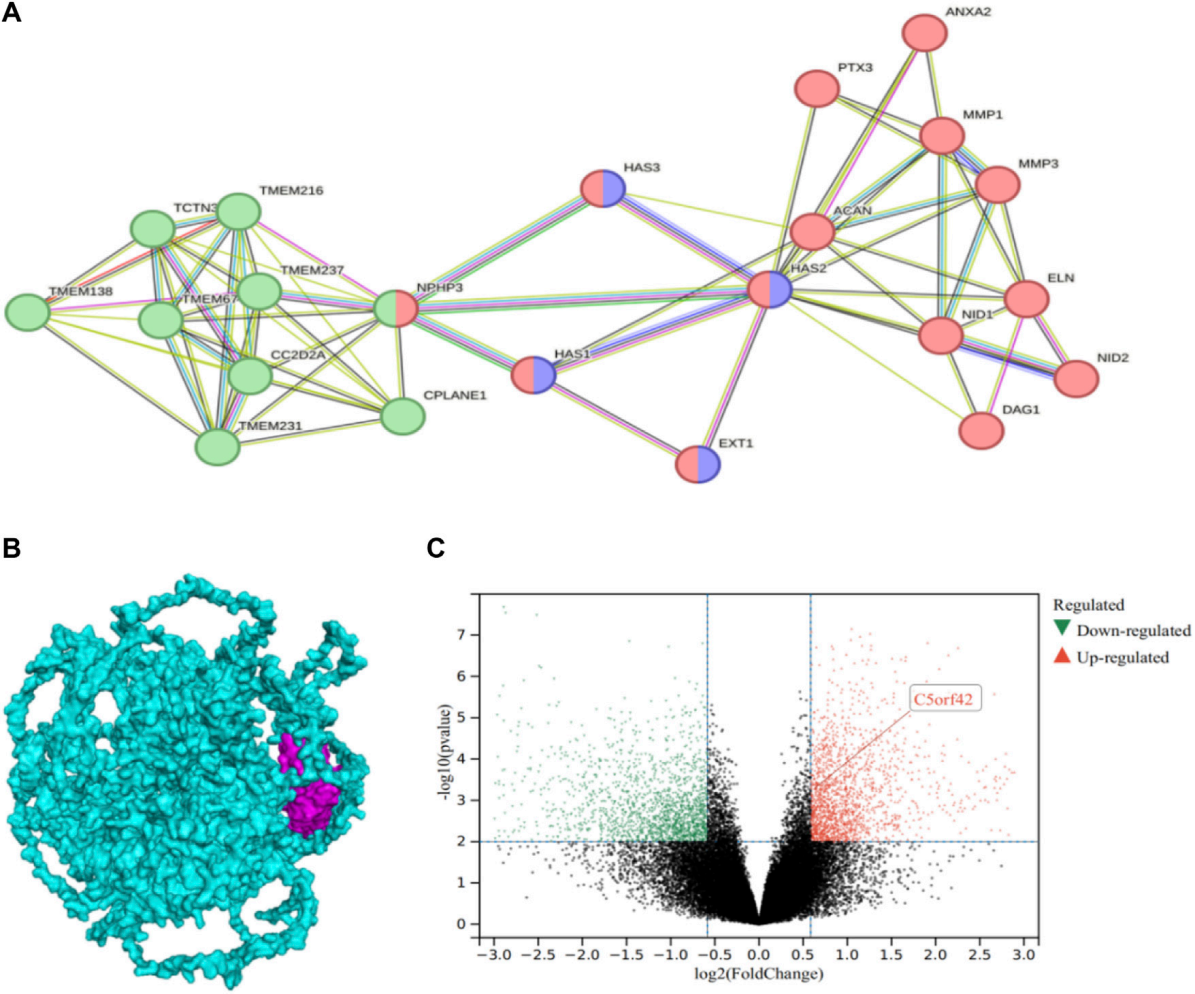
Differential gene expression of *CPLANE1* and analysis of protein-protein interaction networks: **(A)**. Protein-Protein Interaction Networks, PPI: The green node represents cluster 1 extracellular matrix cellular components, the red node belongs to the cilium, and the purple node is involved in the cellular polysaccharide biosynthetic process. **(B)**. The docking of CPLANE1 and NPHP3: Mangenta represents the NPHP3 protein, and blue represents the CPLANE1 protein. **(C)**. Volcano plot of differential gene expression in keratoconus: *CPLANE1* was upregulated in corneal tissue of keratoconus patients compared with the normal control group, and the difference was statistically significant (*p* = 0.000515, <0.05).

Differentially expressed genes (DEGs) according to high-throughput RNA-sequencing data of keratoconus (GSE77938) showed that the level of *CPLANE1* gene expression was upregulated in the corneal tissues of keratoconus patients (experimental group) compared with the normal control group, logFC = 0.62720952, and the difference was statistically significant (*p* = 0.000515, <0.05) (Figure 4C). The PPI network comprised 22 nodes (Figure 4A), with an average node degree of 6.45. The PPI enrichment *p*-value was <1.0e-16, and the local clustering coefficient was 0.819. The Gene Ontology (GO) analysis revealed that those 22 genes belong to cilia genes. Cluster 1 belongs to extracellular matrix (ECM), including ACAN, ANXA2, DAG1, ELN, EXT1, HAS1, HAS2, HAS3, MMP1, MMP3, NID1, NID2, PTX3; Cluster 2 belongs to cilium assembly, including TCTN3, TMEM138, TMEM216, TMEM231, TMEM237, TMEM67, CC2D2A, CPLANE1, and NPHP3, among which EXT1, HAS1, HAS2, and HAS3 belong to the process of cellular polysaccharide biosynthesis. NPHP3 has a dual function in both the cilia and the ECM. As analyzed by ClusPro software, the protein encoded by the *CPLANE1* gene was tightly bound to the NPHP3 protein (Figure 4B), and PPI network analysis showed that CPLANE1-NPHP3 complex proteins played a bridging role between cilia and the ECM.

### 3.6 Literature review

*CPLANE1* is genetically pleiotropic, i.e., mutations result in multiple disease phenotypes, and different phenotypes may occur in different patients. The *CPLANE1* variants associated with Joubert syndrome collected by ClinVar include 37 frameshift mutations, 153 missense mutations, 28 nonsense mutations, 13 splicing site variants, and 25 untranslated region (UTR) variants. This paper summarizes the recently reported pathogenic variants of the *CPLANE1* gene and the associated clinical phenotypes (Table 2).

**TABLE 2:** Pathogenic variation and associated clinical phenotype of the *CPLANE1* gene.

Literature	cDNA change	Protein change	Mutation type	Clinical phenotype
Matoba et al. (2022)	c.3577C>T	Arg1193Cys	missense	The patient presented with an inborn facial anomaly and ataxia, accompanied by a molar tooth sign on a brain MRI. The male patient showed mild intellectual disability, abnormal eye movements, and progressive gait disturbance
	c.3668A>C	Gln1223Pro	missense	
Wang et al. (2023)	c.8948dupT	Pro2984Thrfs*7	frameshift	This patient presented with oculomotor apraxia, dysregulation of breathing pattern, and ataxia. MRI revealed poor continuity of the cerebellum, batwing appearance, and molar tooth sign
	c.247G > T	Gly83X	nonsense	
Zhang et al. (2021a)	c.1270C>T	Arg424X	nonsense	This patient presented with oculomotor apraxia, manifested strabismus, obvious horizontal nystagmus, gaze and gaze tracking impairment, abnormal vestibular eye reflex and light reflex, and distinctive craniofacial abnormalities
	c.8901C>A	Tyr2967X	nonsense	
Xiao et al. (2017)	c.2581G>A	Asp861Asn	missense	This patient presented with cerebellar vermis hypoplasia/dysplasia, oculomotor apraxia, and intellectual disability. A ′molar tooth sign’ was observed through an MRI.
	c.2848C>T	Arg950Ter	nonsense	
Zhang et al. (2021b)	c.1067C>T	Ser356Phe	missense	This research observed different clinical features in two siblings from the same family. The older sibling was classified as a pure JBTS patient, whereas her younger sibling displayed oral-facial-digital defects and was therefore classified as an oral-facial-digital syndrome type VI (OFD VI) patient
	c.8377_8378del	Glu2793Leufs*24	frameshift	
Fei et al. (2022)	c.4459del	Ser1487Valfs*3	frameshift	The proband showed typical clinical features of hereditary JS: brain vermian hypoplasia, hypothalamic hamartoma, lobulated tongue, and eyes with posterior coloboma
	c.7534-14G > A	NA	intronic variant	
Huang et al. (2020)	c.8263dupA	Thr 2,755 Asnfs*8	frameshift	This patient presented with strabismus, horizontal nystagmus, an inability to focus, intellectual impairment, and growth retardation
	c.-47-3C>A	NA	Splice site mutation	

## 4 Discussion

The co-occurrence of Joubert syndrome (JS) and Klinefelter syndrome in the same individual is indeed a rare and unusual finding. Joubert syndrome (JS), a rare neurological disorder first reported by Joubert et al., in 1969 (Joubert et al., 1969). Klinefelter syndrome, one of the most common chromosomal disorders and first described by Klinefelter in 1942, includes multiple karyotypes, of which 47, XXY, is the most common, accounting for about 88%. Postpubertal and adult patients with Klinefelter syndrome are usually characterized by megeoma, long slender limbs, light weight, low body mass index, relatively sparse facial, pubic and armpit hairs, and gynecomastia. In this study, the patient had decreased muscle tone in all four limbs, a finger-to-nose test positive, intentional tremor evident in both hands, a positive Romberg’s sign, instability of gait, level I intellectual disability, poor adaptive behavior, communication disorders, midbrain and cerebellar changes, and a “molar sign” and “bat wing” in the fourth ventricle as shown by cranial magnetic resonance imaging (MRI), thereupon being diagnosed as compound JS. The patient had an unconspicuous prominentia laryngea, sparse beard and facial hair, and learning and language developmental disorders consistent with Klinefelter’s syndrome. JS and Klinefelter’s syndrome have not been reported together in any individual. Both JS and Klinefelter syndrome are systemic diseases, but the skeletal changes are reversed. Most adult patients with Klinefelter’s syndrome tend to have megeoma and have long and slender limbs, but the patient in this case was short in stature with shorter upper and lower limbs. This underscores the complex interplay between genetic factors and phenotypic expression in individuals with multiple coexisting conditions, which manifests as developmental delay and short stature.

JS is a typical example of a cilia-associated disease with significant genetic heterogeneity, and most of the proteins encoded by JS pathogenic genes are found in the cilia or their matrix, which are involved in cilia function and regulation of related signaling pathways, but the specific pathogenic mechanism is unclear. *CPLANE1* is the 42nd open reading frame gene located on chromosome 5, which contains 55 exon regions and encodes a protein inclusive of 3,198 amino acids. As analyzed by prediction software, the CPLANE 1 protein contains 2 coiled-coil domains and may be a transmembrane protein. However, most of the known *CPLANE1* variants reported prior to 2015 are distributed outside the coiled-coil and transmembrane domains (Fei et al., 2022). The Nordic JS cohort study (Romani et al., 2015) showed that the proportion of *CPLANE1* variants is 12% and that *CPLANE1* variants are more likely to cause simplex JS, with the *CPLANE1* gene having an 8.9% mutation rate in simplex JS (Fleming et al., 2017). Renal disease can occur in as many as 1/3 of JS patients, but renal disease is less likely in JS patients with *CPLANE1* variants. The ocular lesions in JS mainly cover oculomotor apraxia (80%), strabismus (74%), nystagmus (72%), and some of which manifest ptosis (43%), retinal degeneration (38%), choroidal retinal defects (30%), and optic nerve atrophy (22%). No correlation was found between the *CPLANE1* gene and the retinal phenotype (Wang et al., 2018). In this study, the genetic test results of the proband showed a *de novo* compound heterozygous variant of *CPLANE1*, which was diagnosed as JS17 in combination with the clinical phenotype. No liver, kidney, or retina were involved in the examination of related organs, and the ocular phenotype did not show oculomotor apraxia or retinal degeneration, but it did show concomitant exotropia and mild ptosis. The cranial MRI showed no other brain malformations except the “molar sign,” which was consistent with the results of previous studies.

In this study, the proband was presented to our hospital with acute keratoconus (KC) in the left eye and keratoconus (completion period, grade 1) in the right eye. However, to date, no literature has reported that KC is a concomitant disease of JC in the available literature. In previous studies of retinal ciliopathies, KC has also been identified as a symptom of various forms of retinal dystrophies as well as systemic syndromes such as Bardet-Biedl syndrome (BBS) (Weihbrecht et al., 2017) and Leber congenital amaurosis (LCA) (Skorczyk-Werner et al., 2020). LCA, the most severe form of retinal ciliopathies, usually occurs shortly after birth and is clinically characterized by severe photoreceptor dysfunction, nystagmus, and a blunted or absent pupil light response (Ruifang et al., 2009; Zou and Sui, 2012). KC, as a symptom of LCA, presents as non-inflammatory degenerative corneal thinning and bulging, leading to further vision loss. LCA patients always press the eyeball, which is usually manifested by excessive eye rubbing, which can accelerate the development of keratoconus. Mutations of the ciliary gene account for about a quarter of the pathogenic genes of LCA, including *CEP164, CEP290, IFT140,* etc., among which *IFT140* gene mutations the disorders of material transport in the photoreceptor primary cilia, which can also lead to LCA with KC (Thompson et al., 2017). Skorczyk-Werner A et al. (Skorczyk-Werner et al., 2020) conducted gene detection and clinical phenotype analysis of 22 LCA families and found that 3 families with ocular features of keratoconus and all with the *CEP290* gene variant had simple LCA and were not complicated with other syndromes. In fact, *CPLANE1* protein is expressed in the cytoplasm and cytoplasmic membranes of many tissues, such as the brain (mainly cerebral cortex), eye (*CPLANE1* mRNA expression detected), endocrine tissues, digestive tract (i.e., colon, the liver, gallbladder, and pancreas), kidneys and bladder, testicles, muscles, connective tissues, skin, bone marrow, and lymphatic tissues. We similarly performed differential expression gene analysis in keratoconus, and the results showed that the *CPLANE1* gene was upregulated in the corneal tissues of keratoconus patients (experimental group) compared with the normal control group, and the difference was statistically significant (*p* = 0.000515, <0.05). The protein encoding the *CPLANE1* gene was tightly bound to the NPHP3 protein, and protein interaction network analysis showed that the CPLANE1-NPHP3 complex proteins acted as a bridge between the cilia and the extracellular matrix tissue. NPHP3 had a dual function in cilia and extracellular matrix tissue, encoding a protein inclusive of the coiled-coil domain (CC), tubulin-tyrosine ligase (TTL) domain, and tetratricopeptide repeat (TPR) domain. The protein encoded by the *CPLANE1* gene interacted with renin, which was required for normal ciliary development and played a role in renal tubule development. Ocular involvement in nephronophthisis (NPHP) caused by *NPHP3* mutations is common, including nystagmus, retinitis pigmentosa, oculomotor nerve disorders, and leber congenital amaurosis, etc. (Chaki et al., 2011). *CPLANE1* also belongs to cilia gene and is involved in the regulation of cilia function and related signaling pathways, but the specific pathogenesis is still unclear. In this study, novel compound heterozygous frameshift variants M1: c.9279dup:*p*.H3094Tfs*18 and M2: c.6515_6522del:*p*.K2172Tfs*37 were detected in the *CPLANE1* gene of the proband, who had ocular phenotypes of keratoconus, exotropia, ptosis, and JS-related signs. The validation by Sanger sequencing showed that M1 was inherited from the mother, M2 from the father, and the younger brother carried M2. The father and younger brother had no ocular phenotypic abnormalities, and the mother’s corneal topography and biomechanical analysis revealed microcorneal morphology in both eyes. Biomechanical examination showed that KC relatives carrying the same pathogenic variant had abnormal corneal morphology, but it was not sufficient for the diagnosis of keratoconus. Therefore, it can be hypothesized that these compound heterozygous variants in the *CPLANE1* gene (*p*.His3094Thrfs*18 and *p*. Lys2172Thrfs*37) are responsible for autosomal recessive KC in this proband. Understanding the underlying genetic mechanisms driving these conditions can provide valuable insights into their pathogenesis and guide personalized treatment approaches.

## 5 Conclusion

In this paper, we report a proband from a Han Chinese family with both *CPLANE1*-Joubert syndrome and X-linked Klinefelter syndrome, as well as the keratoconus phenotype. Our findings suggest that *CPLANE1* variants are more likely to cause KC-Joubert syndrome by affecting the function of the CPLANE1-NPHP3 complex protein, which is considered to act as a bridge between cilia and extracellular matrix (ECM) tissue. The phenotype spectrum of *CPLANE1*-Joubert syndrome may be expanded accordingly. At the same time, it suggests that we also need to pay attention to CNV in the process of genetic testing for monogenic disorders and that the combination of the two can significantly improve the detection rate of pathogenic gene variants associated with diseases. Meanwhile, it was emphasized that genetic testing provides a reliable molecular diagnosis and is crucial in clinical diagnosis and genetic counseling for patients with complex phenotypes.

## Data Availability

The datasets presented in this study can be found in online repositories. The names of the repository/repositories and accession number(s) can be found below: https://www.ncbi.nlm.nih.gov/genbank/, BankIt2820496 PP706089 https://www.ncbi.nlm.nih.gov/, SCV005061709, SCV005061710.

## Supplementary material

SUPPLEMENTARY FIGURE S1 The father's corneal topography map and corneal biomechanics examination: The corneal topography map and corneal biomechanics of both eyes of the father did not show any significant abnormalities.
