# Supplementary figures and images for: Exome sequencing confirms the clinical diagnosis of both joubert syndrome and klinefelter syndrome with keratoconus in a han Chinese family
Source: Front Genet. 2024 Jul 15;15:1417584. doi: 10.3389/fgene.2024.1417584 (PMC11284097; doi:10.3389/fgene.2024.1417584)

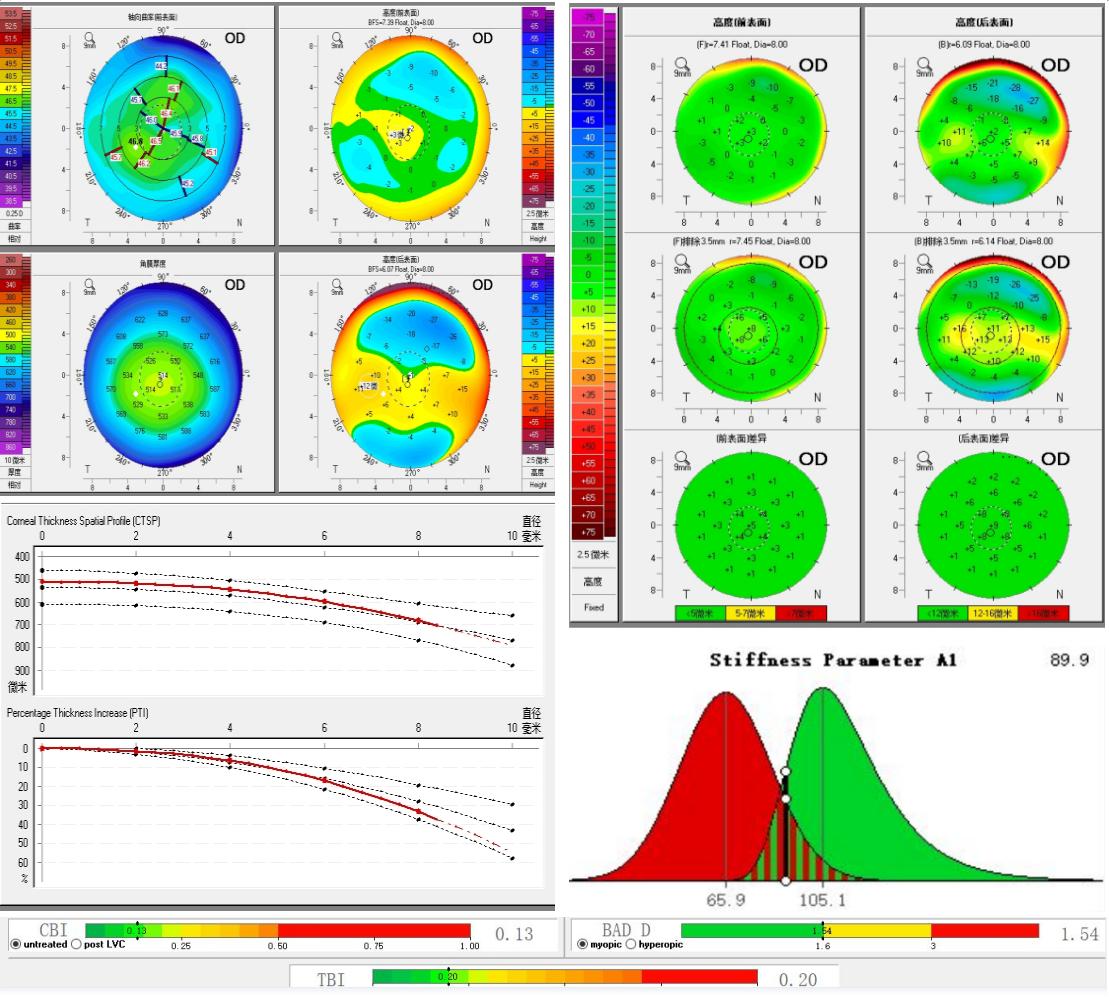

Supplement: Supplementary file 1 [file Image1.JPEG]

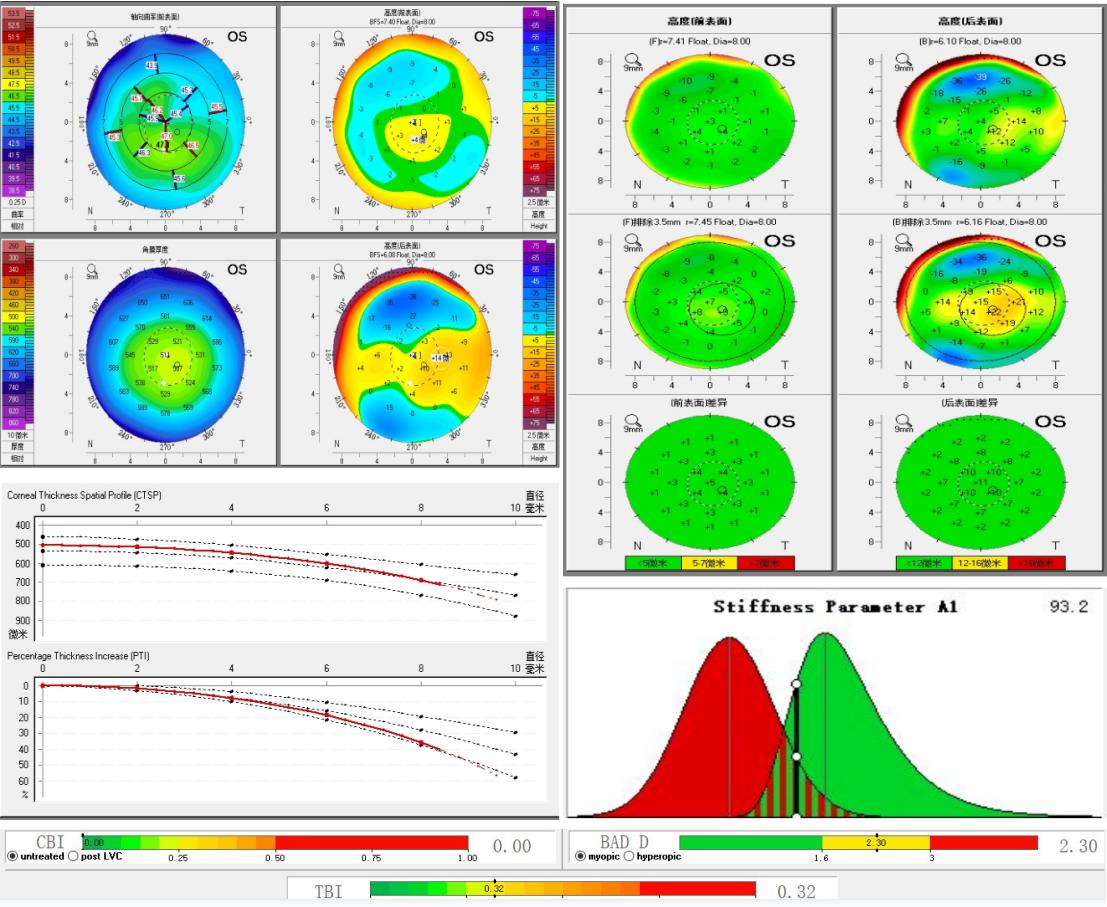

Supplement: Supplementary file 2 [file Image2.JPEG]
